# Urinary Sodium-to-Potassium Ratio Tracks the Changes in Salt Intake during an Experimental Feeding Study Using Standardized Low-Salt and High-Salt Meals among Healthy Japanese Volunteers

**DOI:** 10.3390/nu9090951

**Published:** 2017-08-29

**Authors:** Midori Sasaki Yatabe, Toshiyuki Iwahori, Ami Watanabe, Kozue Takano, Hironobu Sanada, Tsuyoshi Watanabe, Atsuhiro Ichihara, Robin A. Felder, Katsuyuki Miura, Hirotsugu Ueshima, Junko Kimura, Junichi Yatabe

**Affiliations:** 1 Department of Pharmacology, Fukushima Medical University School of Medicine, Fukushima 960-1295, Japan; midorisy@endm.twmu.ac.jp (M.S.Y.); amichacha3@gmail.com (A.W.); takak1209@gmail.com (K.T.); 2 Department of Nephrology, Hypertension, Diabetology, Endocrinology and Metabolism, Fukushima Medical University School of Medicine, Fukushima 960-1295, Japan; sndmak2006@yahoo.co.jp (H.S.); twat0423@fmu.ac.jp (T.W.); 3 Department of Medicine II, Endocrinology and Hypertension, Tokyo Women’s Medical University, Tokyo 162-8666, Japan; atzichi@endm.twmu.ac.jp; 4 Research and Development Department, Omron Healthcare Co., Ltd., Muko 617-0002, Japan; iwahori@belle.shiga-med.ac.jp; 5 Department of Public Health, Shiga University of Medical Science, Shiga 520-2192, Japan; miura@belle.shiga-med.ac.jp (K.M.); hueshima@belle.shiga-med.ac.jp (H.U.); 6 Department of Pathology, University of Virginia Health System, Charlottesville, VA 22908, USA; raf7k@virginia.edu; 7 Center for Epidemiologic Research in Asia, Shiga University of Medical Science, Shiga 520-2192, Japan

**Keywords:** dietary sodium intake, experimental feeding study, salt restriction, standardized diet, urinary sodium-to-potassium ratio

## Abstract

The Na/K ratio is considered to be a useful index, the monitoring of which allows an effective Na reduction and K increase, because practical methods (self-monitoring devices and reliable individual estimates from spot urine) are available for assessing these levels in individuals. An intervention trial for lowering the Na/K ratio has demonstrated that a reduction of the Na/K ratio mainly involved Na reduction, with only a small change in K. The present study aimed to clarify the relationship between dietary Na intake and the urinary Na/K molar ratio, using standardized low- and high-salt diets, with an equal dietary K intake, to determine the corresponding Na/K ratio. Fourteen healthy young adult volunteers ingested low-salt (3 g salt per day) and high-salt (20 g salt per day) meals for seven days each. Using a portable urinary Na/K meter, participants measured their spot urine at each voiding, and 24-h urine was collected on the last day of each diet period. On the last day of the unrestricted, low-salt, and high-salt diet periods, the group averages of the 24-h urine Na/K ratio were 4.2, 1.0, and 6.9, while the group averages of the daily mean spot urine Na/K ratio were 4.2, 1.1, and 6.6, respectively. The urinary Na/K ratio tracked changes in dietary salt intake, and reached a plateau approximately three days after each change in diet. Frequent monitoring of the spot urine Na/K ratio may help individuals adhere to an appropriate dietary Na intake.

## 1. Introduction

It has previously been demonstrated that excess dietary sodium (Na) and insufficient dietary potassium (K) cause blood pressure (BP) elevation [1]. Additionally, epidemiological studies have demonstrated that high dietary Na and low dietary K intakes are also associated with increased cardiovascular disease (CVD) risks [2,3,4,5]. Despite rigorous campaigns to reduce Na and increase K intake, there remains a large discrepancy between the recommended and actual intakes of both Na and K [6,7,8]. Therefore, exact measurement methods are required to facilitate more accurate association studies between dietary salt intake and CVD risk, and avoid discrepant findings arising from technical issues [9,10]. However, the reliable measurement of individual Na and K intake values depends on high-quality, repeated 24-h urine collection (over a period of several days), which is inconvenient and expensive [10,11,12,13,14]. Methods using single spot urine sampling, which are considerably easier than repeated 24-h urine collection, to estimate urinary Na excretion over a single 24-h period have been proposed for the evaluation of population means [15,16,17], but these techniques have inherent problems in terms of accuracy [10,18,19].

The Na/K ratio is considered to be a useful index for use in achieving effective Na reduction and K increase [20]. Epidemiological studies have suggested that the urinary Na/K ratio is a better measurement of dietary Na reduction and K increase in relation to BP and CVD risk assessments than separate Na or K levels [21,22,23,24,25,26,27]. Additionally, the former is easier to measure than the latter, due to its independence from urine collection and creatinine measurement; indeed, repeated Na/K ratio measurements from spot urine samples provide more reliable estimates than those from 24-h urine samples [28,29,30]. Previous studies have demonstrated that single spot urine samples can be used to estimate the population mean values of the 24-h urine Na/K ratio [28], and that four to seven repeated measurements of the Na/K ratio in spot urine samples can be used to estimate the 24-h urine Na/K ratio in individuals [29,30]. Moreover, a recent report on the self-monitoring of the Na/K ratio for both Na reduction and K increase has emphasized that an individual approach can be used; reduction of the Na/K ratio mainly involved Na reduction, while K only changed a little in healthy volunteers recruited from the general population [31].

Although there are separate dietary recommendations for Na and K, currently, no generally accepted target urinary Na/K ratio level has been established [20]. A recent report speculated that a rough estimate of the mean salt intake (g/day) of a population may be derived by calculating approximately two to four times the population mean 24-h urinary Na/K molar ratio in different populations [20]. However, the urinary Na/K ratio levels that correspond to particular Na or K excretion or dietary intake levels have not yet been determined. Additionally, Na and K are excreted through different pathways [13], and the time lapse observed between Na and K intake and their excretion, reflected as the Na/K ratio, remains unknown. Once this is known, the urinary Na/K ratio may be a useful index to monitor when targeting effective Na reduction and K increase [20].

Therefore, we performed this experimental dietary study with the aim of determining a recommended cutoff value for the Na/K ratio for practical salt reduction by monitoring daily Na/K ratios. To this end, we sought to clarify the urinary Na/K ratio corresponding to dietary Na and K intake, and to examine the daily changes in the urinary Na/K ratio after an abrupt change in Na intake, using standardized meals.

## 2. Materials and Methods

### 2.1. Participants

The participants were healthy volunteers without hypertension (defined as an office blood pressure exceeding 140/90 mmHg, at baseline), diabetes, or other known diseases, and who were not taking regular medication. Sixteen individuals were recruited for the study. The study was conducted in accordance with the Declaration of Helsinki, and the Ethics Committee of Fukushima Medical University (Approval No. 1555; Approval Date, 22 Nov. 2012) approved the protocol. All participants provided written informed consent, and no adverse events associated with the study were recorded.

### 2.2. Dietary Protocol

The participants consumed a low-salt (LS) diet (3 g/day sodium chloride (NaCl) = 51 mmol/day) for seven days, which was immediately followed by a high-salt (HS) diet (20 g NaCl/day = 342 mmol/day) for another seven days. The menu plan was designed by three registered dietitians. The mean dietary K intake reported among the Japanese population ranged from approximately 2.2 to 2.8 g/day [32,33]. Thus, we set the K amount to 2.5 g/day (64 mmol/day) throughout the LS and HS diet periods. The K and protein intake amounts were standardized between the LS and HS diets, but the total energy intake was adjusted for each individual. The participants were allowed to consume low-Na, low-K snacks provided to them, but were instructed not to consume food outside of that provided by the study. The meals were cooked from scratch in the test kitchen in accordance with the menu plan. Moreover, all participants gathered in a dining hall for three meals a day during the entire period (both the LS and HS diet periods). Before the LS diet period, and again after the HS diet period, the participants followed their own regular, unrestricted (NS) diet under free-living conditions, without any restrictions on Na or K intake.

### 2.3. Urinary Na/K Ratio and Blood Pressure Measurements

The spot urinary Na/K ratio was measured using a portable personal device throughout the study period (HEU-001-F; Omron Healthcare Co., Muko, Japan; Figure S1a,b) [31], which weighed about 50 g. The device has flat iron electrodes that measure the urinary Na/K ratio, and it displays the result within 1 min. The participants were asked to measure and record their urinary Na/K ratio at every voiding.

### 2.4. Biochemical Analyses of Urine and Blood

On the last day of each diet period, including the NS diet period before commencing the LS diet period, 24-h urine collections were conducted, in addition to spot urine Na/K ratio measurements. Participants were instructed to collect all urine samples using a measuring cup, with all urine saved in 2 L bottles, unless urine collection was unsuccessful or contaminated with feces. To avoid under- and overcollection, the start and end times of the 24-h urine collection were supervised by clinic staff. The 24-h urine collection began with the participants voiding urine to empty their bladder during the first visit and finished during the visit on the following day when the participants collected their urine in the bottles on-site and returned with their 24-h urine collection bottles. Urine was collected for 24 h and kept cold during the collection period. The samples were handled by certified staff. The Na, K, creatinine, and plasma aldosterone concentrations and plasma renin activity were determined in these samples, as described previously [34].

### 2.5. Statistical Analysis

#### 2.5.1. Dietary Intake vs. Urinary Excretion

Participants obtained a fixed amount of dietary Na and K from the controlled meals provided during the study, and the Na/K ratio, and Na and K excretions measured from the urine were compared in order to clarify the relationship between dietary Na intake and the urinary Na/K molar ratio. All *p* values were two-sided, and *p* values < 0.05 were considered to indicate statistically significant differences. Statistical analyses were performed using IBM SPSS Statistics (IBM Japan, Tokyo, Japan). The differences between the groups were analyzed using one-way repeated-measure analysis of variance with Bonferroni correction or the Friedman test, where applicable. Data are presented as mean ± standard deviation (SD), unless otherwise noted.

#### 2.5.2. Daily Na/K ratio Assessment from Multiple Spot Urine Samples for Estimating the 24-h Urine Na/K Ratio

Since participants were consuming a controlled diet in this study, the day-to-day variability of Na and K were expected to be minimized. Thus, we used a single 24-h urine sample and daily repetitive measurements of the spot urine Na/K ratio to evaluate the dietary levels of Na and K for each individual in this study. Iwahori et al. previously proposed repetitive measurements of the spot urine Na/K ratio sampling from different days to estimate the seven-day 24-h Na/K ratio [29,30]; however, daily repetitive spot urine Na/K ratio sampling from the same day has not yet been established for estimating a single-day 24-h urine Na/K ratio under controlled dietary conditions. Thus, this estimation method was additionally evaluated in this study. Spearman’s rho values for Na/K ratios were calculated to examine the correlation between the daily mean values of the spot urine Na/K ratio and the corresponding values for 24-h urine specimens. Agreement between the daily mean values of the spot urine Na/K ratio and the 24-h urine Na/K ratio was examined using the Bland-Altman method [35]. Bland-Altman plots showing the daily means of the spot urine Na/K ratio and the 24-h urine Na/K ratio values versus the difference between these two values, were used to assess the mean difference (bias). The upper and lower agreement limits (mean difference ± 1.96 × SD of difference) between the daily means of the spot urine Na/K ratio and the 24-h urine Na/K ratio were calculated, as was the difference between the upper and lower agreement limits (defined as 95% limit of the difference).

## 3. Results

### 3.1. Baseline Parameters of the Participants

Table 1 shows the baseline parameters of the participants on an unrestricted diet. We performed the study from January to February 2014. Excluding two participants who dropped out for personal reasons, 14 eligible individuals, comprising five men and nine women, aged 21 to 26 years, completed the study and their data were included in the final analysis.

### 3.2. Changes in 24-h Urinary Na/K Ratio and Basic Parameters During the Study

Data from urine samples with a low creatinine content (<0.8 g/day or <70% of maximal urinary creatinine excretion) were excluded from the analysis. The average urinary Na/K molar ratios of the 14 participants, which was calculated from 24-h urine collection, were 4.2 ± 1.9, 1.0 ± 0.3, and 6.9 ± 1.5, (mean ± SD) on the last day of the NS, LS (3 g/day), and HS (20 g/day) diet periods, respectively. Their average dietary NaCl levels, based on urinary excretions calculated from 24-h urine collection, were 7.3, 1.7, and 17.1 g/day for the NS, LS, and HS diet periods, respectively. Additionally, the urinary K excretion level was 1.16 g/day for the NS diet period. The NaCl excretion percentages, calculated based on Na excretion from 24-h urine for the LS and HS diet periods, were 57% and 86%, respectively, of the designed dietary NaCl content of each respective diet. The urinary K excretion level increased by 33% during the HS diet period, even though a constant dietary K intake was maintained. Furthermore, the urinary K excretion percentages were 48% and 66% of the designed dietary K content for the LS and HS diets, respectively, under the assumption that 90% and 70% of dietary Na and K, respectively, would be excreted in the urine.

No significant differences in blood pressure between the three diet periods were observed (Table 2). Hematocrit was significantly reduced during the HS diet period. Additionally, the urine volume increased by 1.5-fold, but the plasma renin activity significantly decreased during the HS diet period as compared to the LS diet period.

### 3.3. Changes in Self-Measured Urinary Na/K Ratio During the Study

**Figure 1:**
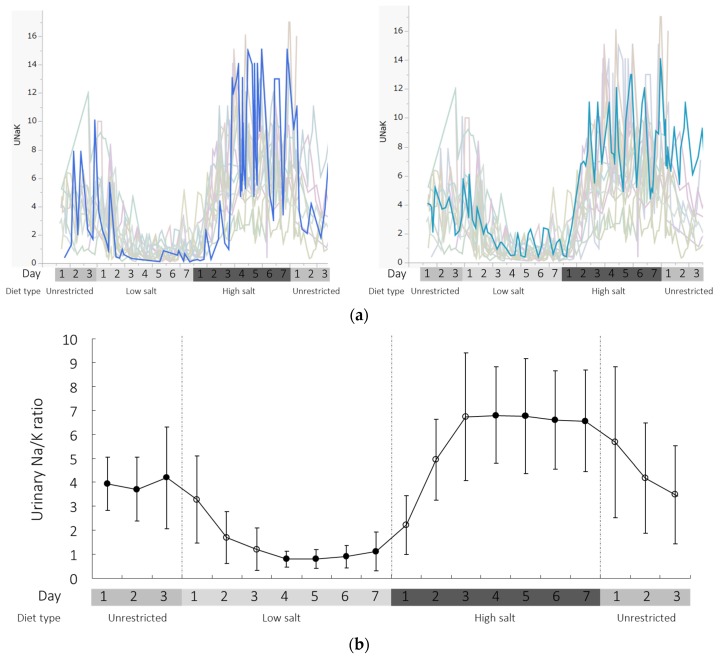
Time-dependent changes in urinary Na/K ratio (UNaK) during unrestricted, low-salt (3 g NaCl/day), and high-salt (20 g NaCl/day) diet periods measured by the device. (**a**) Individual trends and (**b**) group average throughout the study.

In this study, the group means of the urinary Na/K ratio obtained from the daily mean of repetitive spot urine Na/K ratio determinations were 4.2 ± 2.1, 1.1 ± 0.8, and 6.6 ± 2.1 (mean ± SD) on the last day of the NS, LS (3 g/day), and HS (20 g/day) diet periods, respectively. The individual spot urine Na/K ratio fluctuated under the controlled diets, with the variation being large during the HS diet period, but small during the LS diet period (Figure 1a). Diurnal variation was readily observed in most participants during the HS diet period. The group average urinary Na/K ratio clearly showed a plateau approximately three days after the change in the dietary Na level (Figure 1b). In individual traces, the time to reaching the plateau varied between three and four days (Figure 1a).

The daily mean range of the device-measured individual urinary Na/K molar ratio was narrow during the LS diet period (0.23–2.80), but wide during the HS diet period (3.16–10.14). In fact, 74% and 94% of the 207 individual-based urinary Na/K molar ratio measurements taken during days four to seven of the LS diet were ≤1.0 and ≤2.0, respectively. During the last four days of the HS diet, 7.6%, 42.6%, 31.4%, 29.4%, and 15.2% of the 278 individual-based urinary Na/K molar ratio measurements were ≤4.0, 4.1–6.0, 6.1–8.0, 8.1–10.0, and >10, respectively.

### 3.4. Validation of Daily Mean Spot Urine Na/K Ratio for Estimating 24-h Urine Na/K Ratio

**Figure 2:**
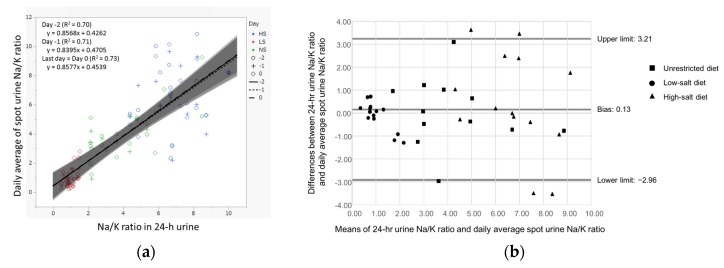
Correlation and agreement analyses between individual daily means of the spot urine Na/K ratio and 24-h urine Na/K ratio: (**a**) Spearman’s correlation between the 24-h urine Na/K ratio during the last day of each diet period and individual daily means of the spot urine Na/K ratio of the last three days of each diet period. Data points from unrestricted (NS), low-salt (LS), and high-salt (HS) diet periods are shown in green, blue, and red, respectively. The regression lines calculated for the last day (0), the day before (−1), and two days before the last day (−2) are shown with 95% confidence interval in gray areas. (**b**) Bland-Altman plot of the 24-h urine Na/K ratio and daily means of the spot urine Na/K ratio on the last day of each diet period.

The individual daily means of the spot urine Na/K ratio for the last three days of each diet period correlated well with the 24-h urine Na/K ratio of the last day of each diet period (Figure 2). For the daily mean spot urine Na/K ratio determined on the last day of each diet period, the bias estimate, defined as the difference between the Na/K ratios of the 24-h and spot urine collections, was 0.13, whereas the 95% limit of the difference ranged from −2.96 to 3.21, according to the Bland–Altman method (Figure 2). The voiding frequency ranged from three to eight times per day (mean: 4.0 and 5.0 times per day during the LS and HS diet periods, respectively) in this study. Figure S2 shows the correlations between the 24-h urine Na/K ratio on the last day and the means of different numbers of randomly selected spot urine Na/K ratios during the last three days of each diet period. The correlation coefficient ranged from 0.74 to 0.88, the bias ranged from 0.16 to 0.39, and the 95% lower and upper limits of difference ranged from −0.45 to −0.19 and 0.72 to 1.1, respectively, among these combinations. The correlation between the mean Na/K ratio of three to seven spot urine samples and the 24-h urine collection remained high, and their agreement was good (Figure S2).

## 4. Discussion

The relationship between the urinary Na/K ratio and dietary Na/K ratio, Na, and K levels has been unclear. Frequent monitoring of the urinary Na/K ratio during acute changes in salt intake has not been reported previously. This nonparallel, experimental dietary study demonstrated the relationship between standardized dietary Na and K intake levels and the corresponding urinary Na/K ratio among healthy volunteers. Association studies have demonstrated the benefits of monitoring the Na/K ratio to assess Na reduction and K increase in relation to BP and CVD risks, as compared with monitoring Na and K levels separately. However, the urinary and dietary Na/K ratio levels used for assessing risk scores differed among these previous studies due to the percentage of diet reflected in the urine [25,26,27,36,37]. In the present study, the urinary Na/K ratio tracked the changes in dietary Na intake; changes were reflected in the urinary Na/K ratio within approximately three days under standardized dietary conditions. Furthermore, the daily means of the urinary Na/K ratio correlated well with those of the dietary Na/K ratio, and the former was approximately 1.3 times the value of the latter.

Use of formulas to estimate 24-h urinary Na excretion from a single spot urine sample depends on other parameters, such as body weight and creatinine clearance [15,16,17], and may be biased [18,19], whereas the Na/K ratio is independent of both creatinine excretion and body weight, and is also unbiased [26]. Repeated spot urine Na/K ratios may therefore be a useful and practical means for obtaining individual values of the urinary Na/K ratio [29,30]. The spot urine Na/K ratio showed higher correlations and better agreements with 24-h urine values than the Na or K level alone [28,29,30], especially when using repeated spot urine measurements [29,30]. The results of the agreement and correlation analyses of four to seven repeated measurements of the spot urine Na/K ratio were similar to those of one to two-day 24-h urine Na/K ratios, when compared with the gold standard seven-day 24-h urine Na/K ratio [29,30].

In this study, we primarily used the daily mean of the spot urine Na/K ratio, but we also examined the use of different repetitions of spot urine sampling to determine their agreement with the 24-h urine Na/K ratio under standardized and unrestricted dietary conditions. Increasing the number of random spot urine Na/K ratio samples is known to improve the correlation with the seven-day 24-h urine Na/K ratio [29,30], as confirmed by our findings, although the level of agreement in our study was slightly less than that reported previously. This may be because only a single 24-h urine sample collection was performed for each dietary condition in the present study. However, the correlation data indicated that the mean of multiple spot urine Na/K ratios reflected the 24-h Na/K ratio well. The daily mean of the spot urine Na/K ratio was roughly equivalent to the mean of five random spot urine Na/K ratio measurements. This may be explained by voiding frequency. For relatively short-term studies, the daily mean spot urine Na/K ratio measured at least four days after a change in diet may serve as a simple substitute for a 24-h urine Na/K ratio.

In this regard, a self-monitoring device for the urinary Na/K ratio measurement, which provides prompt on-site feedback, has been evaluated with a view to supporting an individual approach for Na reduction and K increase [31]. Repeated measurement of the spot urine Na/K ratio may be a low-burden method for monitoring adherence to World Health Organization (WHO) guidelines on Na and K intake. However, interpretation of the individual estimate obtained through the repeated spot urine Na/K ratio measurement is difficult, because the urinary Na/K ratio corresponding to the dietary Na and K intake has not yet been reported. Moreover, a formally recommended cutoff value for this ratio has not been established to date. Our findings provide a basis for Na/K monitoring, which is a prerequisite for setting such goals.

Rakova et al. have demonstrated that individual urinary Na excretion fluctuated under controlled dietary conditions, which was similar to the results of our study [14]. However, the amount of dietary salt intake correlated well with the group mean 24-h urinary Na/K ratio, based on a 24-h sample collected during the last day of each period, and the urinary Na/K ratio obtained by determining the daily mean of repetitive spot urine Na/K ratio measurements at least three days after changing the standardized diet. Thus, the single-day 24-h urine collection on the last day of LS and HS diet periods reasonably represented the group estimate of Na intake during these periods, as the day-to-day variation in Na excretion was minimized by means of the standardized diets. Additionally, the urinary Na/K ratio is known to show diurnal variation [38]. Thus, daily repetitive spot urine Na/K ratio sampling may have corrected the effect of diurnal variation and therefore show a good association with dietary salt intake and the 24-h urine Na/K ratio under controlled dietary conditions.

We determined the urinary Na/K molar ratio corresponding to the Na and K intake using our standardized diets. The dietary Na/K molar ratios in this study were designed to be 0.80 and 5.3 on the LS (NaCl, 3 g/day; K, 2.5 g/day; Na/K molar ratio = 51.3/64.1 mmol/mmol) and HS (NaCl, 20 g/day; K, 2.5 g/day; Na/K molar ratio = 341.9/64.1 mmol/mmol) diets. Previous reports have indicated that 80–95% of dietary Na [13,39,40,41,42] and 63–77% of dietary K [13,40,41] is excreted in the urine. Thus, if 90% and 70% of dietary Na and K, respectively, are excreted in the urine, the urinary Na/K ratio would be 1.3 times the dietary Na/K ratio, which indicates that the theoretical urinary Na/K molar ratio should be 1.0 and 6.8 on the LS and HS diets, respectively, in the present study. The actual group averages of the 24-h urine Na/K molar ratio were 1.0 and 6.9 on the LS and HS diets, respectively; the urinary Na/K molar ratio obtained as the daily mean of spot urine Na/K ratios were 1.1 and 6.6, which were close to these theoretical values. However, the 24-h urine Na and K excretions were 58–81% and 48–66%, respectively, of the designed dietary content during the LS and HS diet periods.

As shown in Table 2, the component of the urinary Na/K ratio that showed the predominant change was the amount of Na excreted, which reflected NaCl consumption. Although there was a significant increase in urinary K excretion during the HS diet period, which may have resulted from increased Na delivery to the distal tubules of the kidney [43,44], the change in K was much smaller than that in Na, and K excretion did not differ between the HS and the NS diet periods. Moreover, a recent self-monitoring intervention study on lowering the urinary Na/K ratio mainly resulted in Na reduction, with little change observed in K [31]. These findings suggest that the change in urinary Na/K ratio in real world conditions (with Na and K intake levels close to the average of the general population) would be mainly due to changes in Na, and would be relatively independent of K. However, these assumptions may not be applicable to individuals with an extreme K intake or to patients with kidney diseases. Furthermore, a decrease in urinary Na excretion during the LS diet period may have been due to sustained extra-renal Na loss, and the lower-than-expected Na and K excretion seen throughout the study may have been due to the loss of Na in sweat, as some participants participated in sports during the study, in addition to possible incomplete urine collection. Considering that 24-h urine Na and K measurements may result in Na and K intake underestimations due to incomplete collections [10], the use of the Na/K ratio is more advantageous than separate Na or K excretion assessments, because it is resistant to systematic errors related to urine volume. The finding that the rate of K excretion was lower than that of Na excretion in our study may also be explained by a relative K deficiency in our study participants and due to racial differences in the K balance [40].

Changes associated with salt intake were appropriately observed in this study, such as those in urine volume, voiding frequency, serum creatinine levels, hematocrit, plasma aldosterone concentrations, and plasma renin activity. No significant changes in blood pressure were observed in our study, which may be due to the inclusion of young normotensive participants and a short salt-loading duration. The normotensive population has been reported to display a lower percentage of blood pressure salt-sensitivity than the hypertensive population [45].

Dietary NaCl intake may be roughly estimated from the urinary Na/K ratio by examining the population mean 24-h urine Na/K molar ratio and the population mean 24-h urine salt (NaCl) (g/day) concentration [30]. In this study, the ratio of dietary NaCl (g/day) to urinary Na/K molar ratio was approximately 3, in different dietary conditions; the group mean 24-h urine Na/K molar ratio during LS and HS diet periods were 1.0 and 6.9, when the group mean salt intake was 3 (g/day) and 20 (g/day), respectively. Findings from the INTERMAP (International study of macro- and micro-nutrients and blood pressure) study showed that the population mean dietary salt intake of 11.8 (g/day) corresponded to a population mean 24-h urine Na/K molar ratio of 4.3, in four Japanese cohort centers [32]. Thus, a rough estimate of the group mean salt intake (g/day) may be taken as being approximately three times the group mean 24-h urinary Na/K molar ratio. Thus, considering that the group mean urinary Na/K molar ratio during the NS diet period was 4.2, the group mean salt intake may have ranged from 12 to 13 g/day.

WHO reports suggest that guideline-targeted Na and K intake levels would yield a Na/K ratio of approximately 1.0 [46,47]. Extrapolating from the present study, an average urinary Na/K molar ratio of approximately 1 would correspond to a dietary salt intake of 3 g per day, rather than 5 g per day. However, given that the K intake level during the LS period in our study was lower than that recommended by the WHO guideline, the dietary salt intake level needed to satisfy the Na/K ratio of 1 should be lower than the Na level recommended by the WHO guideline. This phenomenon may occur in a real-world setting. During the LS diet period, more than 70% and 90% of spot urine Na/K molar ratio measurements were ≤1.0 and ≤2.0, respectively. Considering that Cook et al. reported that a Na/K molar ratio of between 1 and 2 resulted in the lowest CVD risk [25], a urinary Na/K ratio of <2 might be a reasonable current goal for most individuals, in terms of lowering BP and reducing CVD risk. Further investigations are needed to explore the possibility that a lower target than that currently aspired to might be optimal.

This study has several limitations. First, the participants were young Japanese individuals, and the sample size was relatively small. Second, it is not known whether the urinary Na/K ratio acclimatizes over a longer period after an acute change in diet. Another limitation of the study is that the actual food intake and data collection were dependent on the participants, although we attempted to achieve high-quality 24-h urine collection in this study by monitoring the start and end of the collection period [10,48]. However, the urinary excretion-to-dietary intake ratio of electrolytes were somewhat lower than those previously reported [13,14,39,40,41,42]. The relatively low NaCl and K excretion values and the low urine volume may be partly due to incomplete urine collection, as well as to excess Na loss in sweat during sports participation.

## 5. Conclusions

In conclusion, we determined that the group average of the individual daily means of the spot urine Na/K ratio tracked the changes in dietary levels of Na. Frequent urinary Na/K ratio monitoring may be a useful index of individuals’ dietary Na and K levels, which may help individuals to adhere to appropriate Na intake amounts, and thereby may contribute to the reduction of cardiovascular complications.

## Figures and Tables

**Table 1:** Baseline parameters of the participants on an unrestricted diet.

Parameter	Unit	Mean ± SD (Range)
Men/women		5:9 = 35.7%
Age	Years	22.5 ± 0.3 (21–26)
Weight	kg	53.3 ± 5.1 (44.9–63.1)
BMI	kg/m^2^	20.4 ± 2.0 (17.2–25.6)
Fasting serum glucose	mg/dL	83 ± 7
Insulin	μIU/mL	7.2 ± 2.6
Morning pulse rate	Beats per min	66 ± 12
Evening pulse rate	Beats per min	65 ± 10

BMI, body mass index; SD, standard deviation.

**Table 2:** Parameters measured at the end of each diet period.

Parameter	Unit	Unrestricted	Low Salt	High Salt	*p* Value
Morning SBP	mmHg	104 ± 9	103 ± 7	102 ± 11	0.769
Morning DBP	mmHg	69 ± 7	65 ± 8	70 ± 7	0.102
Morning MBP	mmHg	76 ± 15	78 ± 7	81 ± 7	0.341
Morning pulse rate	/min	66 ± 12	67 ± 13	64 ± 9	0.652
Hematocrit	%	44.3 ± 3.3	44.4 ± 3.9	42.7 ± 3.0 *	0.045
PAC	pg/mL	208 ± 97	391 ± 204 ^$^	133 ± 63 *^,#^	0.001
PRA	ng/mL/h	1.3 ± 1.1	2.9 ± 1.5 ^$^	1.0 ± 1.4 *	0.006
Creatinine	mg/dL	0.69 ± 0.13	0.71 ± 0.14	0.65 ± 0.11 *^,#^	0.000
Serum Na	mEq/L	141 ± 2	141 ± 1	141 ± 2	0.220
Serum K	mEq/L	4.0 ± 0.2	4.0 ± 0.2	4.1 ± 0.3	0.940
Urine volume	mL/day	926 ± 369	978 ± 336	1416 ± 495 *^,#^	0.016
Urine Na	mmol/day	128 ± 47	30 ± 7 ^$^	281 ± 42 *^,#^	0.001
Urine K	mmol/day	32.3 ± 9.4	29.5 ± 6.0	39.2 ± 7.6 *	0.032
Urine creatinine	g/day	1.20 ± 0.25	1.14 ± 0.20	1.23 ± 0.29	0.093
Morning SBP	mmHg	104 ± 9	103 ± 7	102 ± 11	0.769
Morning DBP	mmHg	69 ± 7	65 ± 8	70 ± 7	0.102

SBP, systolic blood pressure; DBP, diastolic blood pressure; MBP, mean blood pressure; PAC, plasma aldosterone; PRA, plasma renin activity. Mean ± SD. For urinary data, those with urinary creatinine excretion of <0.8 g/day or <70% of the maximal urinary creatinine excretion were excluded from the analysis (*n* = 11–12). ^$^ *p* < 0.05 for unrestricted versus low-salt diet. * *p* < 0.05 for low-salt versus high-salt diet. ^#^ *p* < 0.05 for unrestricted versus high-salt diet.

## Supplementary Materials

The following are available online at www.mdpi.com/2072-6643/9/9/951/s1. Figure S1: (a) Urinary Na/K ratio monitor (HEU-001F, Omron Healthcare Co., Ltd., Kyoto, Japan); (b) Pearson’s correlation coefficient for the urinary Na/K ratios measured by the portable urinary Na/K ratio monitor (HEU-001F, Omron Healthcare Co., Ltd., Kyoto, Japan) and by a biochemical inspection center (BML Inc., Tokyo, Japan). Figure S2: Correlation between one random spot urine Na/K ratio as well as the average of three, five, or seven random spot urine Na/K ratios obtained during the last three days of each diet period and the 24-h urine Na/K ratio obtained on the last day of each diet period.
