# Supplementary material for: Urinary Sodium-to-Potassium Ratio Tracks the Changes in Salt Intake during an Experimental Feeding Study Using Standardized Low-Salt and High-Salt Meals among Healthy Japanese Volunteers
Source: Nutrients. 2017 Aug 29;9(9):951. doi: 10.3390/nu9090951 (PMC5622711; doi:10.3390/nu9090951)
Supplement: Supplementary file 1 [file nutrients-09-00951-s001.pdf]

Supplementary Material:

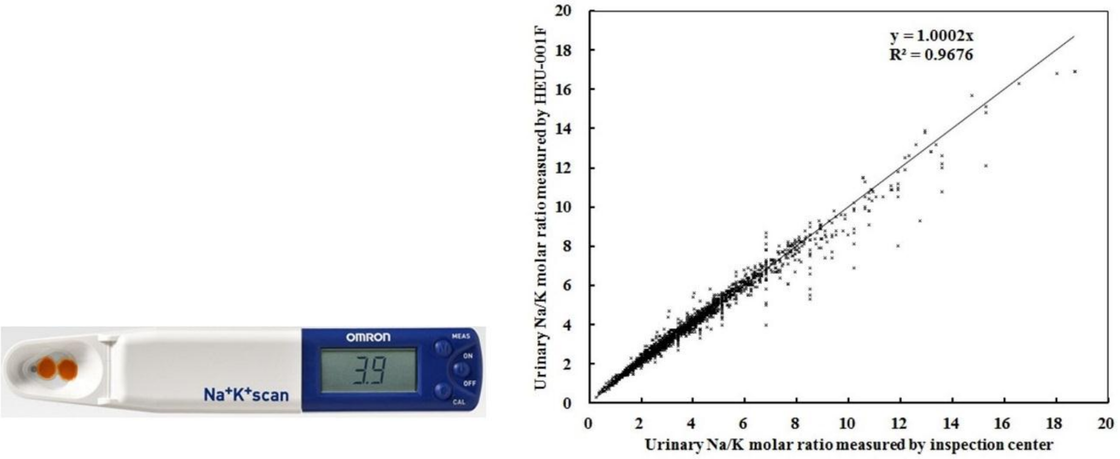

(**a**) (**b**)

Figure S1. (**a**) Urinary Na/K ratio monitor (HEU-001F, Omron Healthcare Co., Ltd., Kyoto, Japan). (**b**) Pearson's correlation coefficient for the urinary Na/K ratios measured by the portable urinary Na/K ratio monitor (HEU-001F, Omron Healthcare Co., Ltd., Kyoto, Japan) and by a biochemical inspection center (BML Inc., Tokyo, Japan).

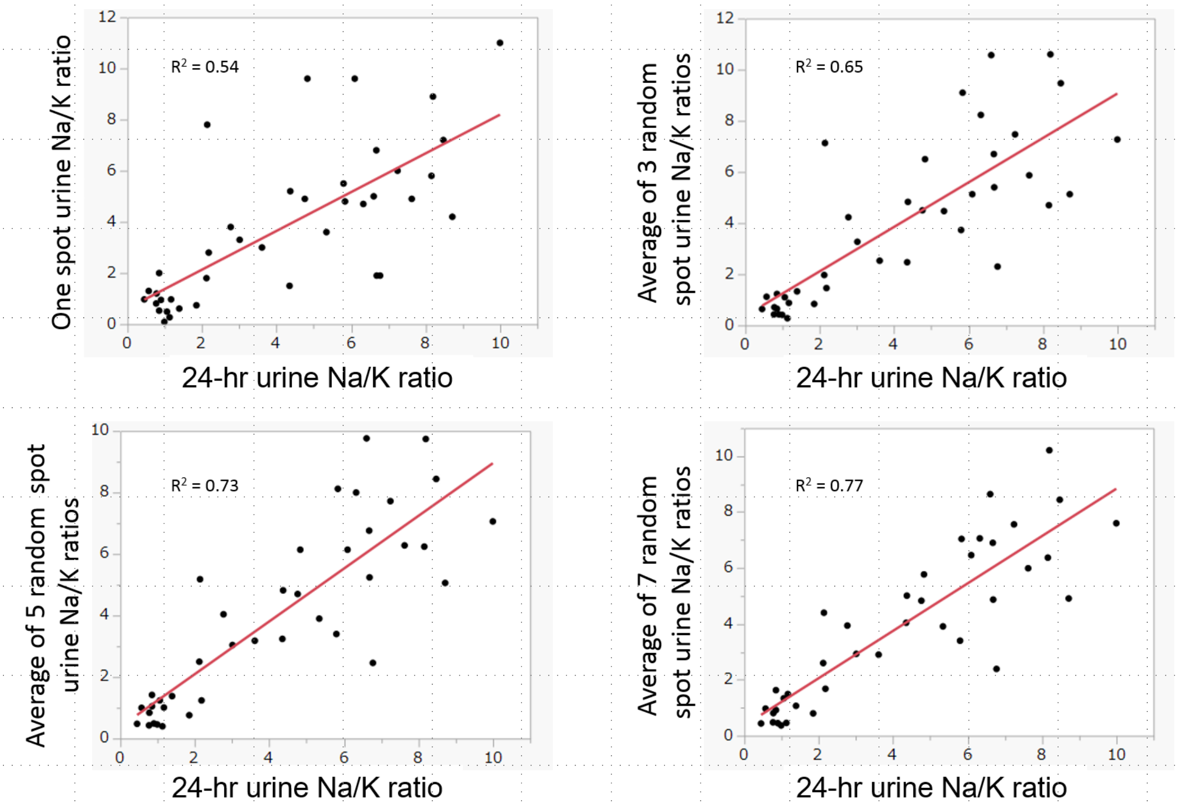

Figure S2. Correlation between one random spot urine Na/K ratio as well as the average of three, five, or seven random spot urine Na/K ratios obtained during the last three days of each diet period and the 24-h urine Na/K ratio obtained on the last day of each diet period. $p < 0.001$ for all correlations.
